# Esophageal Candidiasis in a Non-HIV Patient: A Primary Care Diagnosis

**DOI:** 10.7759/cureus.24312

**Published:** 2022-04-20

**Authors:** Sofia Rodrigues, Vera Leitão Esteves, Teresa G Martins

**Affiliations:** 1 General Practice and Family Medicine, Unidade de Saúde Familiar (USF) Descobertas, Lisboa, PRT; 2 General Practice and Family Medicine, Unidade de Saúde Familiar (USF) Descobertas, Lisbon, PRT; 3 General Practice and Family Medicine, Unidade de Saúde Familiar (USF) Monte Pedral, Lisbon, PRT

**Keywords:** proton pump inhibitors, inhaled corticosteroid, immunocompetent, diabetes mellitus type 2, esophageal candidiasis

## Abstract

A 74-year-old man visited his family doctor for dysphagia and was diagnosed with esophageal candidiasis. His risk factors included type 2 diabetes mellitus, long-term intake of budesonide/formoterol inhaler 160/45 µg, and pantoprazole 20 mg. He was treated with fluconazole 200 mg per day for 14 days. Other factors of immunosuppression were excluded, and his chronic medication was adapted by starting him with a proton pump inhibitor withdrawal plan and switching his inhaled device to a formoterol-only device without an inhaled corticosteroid. The patient had complete remission of the symptoms on the seventh day of treatment without relapse to date. The key point is that iatrogenic factors should be considered in the presence of esophageal candidiasis in immunocompetent patients and a therapeutic review is an important tool that should be used in every primary care appointment to refrain from long-term prescriptions without clinical indication and, consequently, to avoid adverse events.

## Introduction

Candida esophagitis is a common opportunistic infection in immunocompromised hosts, being usually common among patients infected with human immunodeficiency virus (HIV). Nonetheless, at the present time, candida esophagitis in non-HIV patients has been increasing [1]. The main risk factors in immunocompetent hosts are hematologic malignancies, hematopoietic cell transplant, solid organ cancer, and cytotoxic chemotherapy. Other risk factors include comorbidities such as diabetes mellitus, peptic ulcer disease, and tobacco use. It is also very important to consider iatrogenic risk factors in the presence of candida esophagitis in immunocompetent patients, most commonly secondary to long-term use of inhaled corticosteroids, such as fluticasone propionate or budesonide [2,3], long-term treatment with antibiotics [4], and prolonged use of proton pump inhibitors [5].

## Case presentation

A 74-year-old man visited his family doctor for a three-day course of dysphagia, described as a "pressure when trying to swallow" appearing some seconds after ingestion and that was referred mainly to the chest rather than the neck, for both solids and liquids. He denied weight loss, nausea and vomiting, odynophagia, abdominal pain, hematemesis, fever, and night sweating. He also denied that its presentation was abrupt or right after ingestion of any type of food or drinks. He had no respiratory symptoms. Physical examination, including oropharyngeal, cervical and abdominal inspection, auscultation, percussion, and palpation, did not present relevant findings as well as a summary of neurologic and skin examination.

His past medical history consisted of type 2 diabetes mellitus diagnosed ten years ago, well-controlled since 2015, with glycosylated hemoglobin (HbA1c) below 6.5%. He also had gout, hypertension, hip osteoarthritis, renal lithiasis, chronic pulmonary obstructive disease, benign prostatic hyperplasia, and erectile dysfunction (that could be a complication of diabetes mellitus). He denied a smoking history. The patient was medicated with (presented in a total dose per day): metformin 1,700 mg and sitagliptin 100 mg, indapamide 1.5 mg, valsartan 160 mg, lercanidipine 20 mg, tansulosine 0.4 mg, atorvastatin 20 mg, budesonide/formoterol inhaler 160/45 µg, alendronic acid 10 mg and with pantoprazole 20 mg, the last one for the last 25 years for a peptic ulcer that had never relapsed.

**Figure 1:**
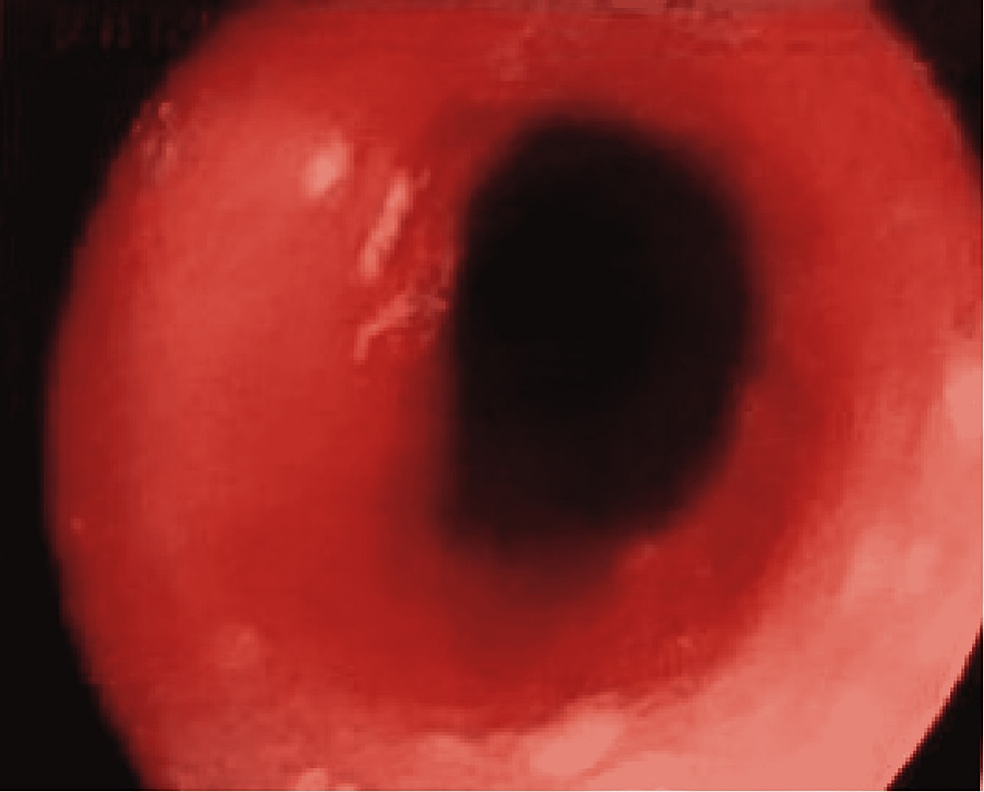
Endoscopic image of the upper thoracic esophagus Whitish plaques partially covering the esophagus are visible at three, six, and ten o'clock.

Esophagogastroduodenoscopy revealed whitish plaques suggestive of esophageal moniliasis mainly in the first half of the esophagus (Figure 1), which was not biopsied. Regarding the hypothesis of esophageal candidiasis, it was important to exclude any type of immunocompromise and malignant disease. The blood tests (hemogram, leucogram, platelet count and peripheral blood smear, serum protein electrophoresis, c-reactive protein, sedimentation rate, serologies for HIV, hepatitis B, and C) and the image exams (computed tomography scan of thorax, abdomen, and pelvis) showed no relevant changes [6].

Esophageal candidiasis was presumed, and oral fluconazole 200 mg per day was initiated and maintained for 14 days [1]. His chronic medication was modified, starting with a pantoprazole withdrawal plan and exchanging his inhaled device for a formoterol-only device since the patient had no indication for inhaled corticosteroids. The patient was reevaluated one week after the beginning of fluconazole, on the 14^th^ day of treatment, posteriorly one month later, and then every six months. He noted complete resolution of symptoms on the seventh day without relapse to the present day.

## Discussion

We report a case of esophageal candidiasis in a non-HIV patient. The main risk factors for the development of esophageal candidiasis, in this case, are type 2 diabetes mellitus and iatrogenic risk factors, namely the prolonged use of proton pump inhibitors and inhaled corticosteroids. Immunosuppression, caused either by type 2 diabetes mellitus and by the use of inhaled corticosteroids, or varying degrees of hypochlorhydria, potentially caused by prolonged treatment with proton pump inhibitors, may have synergically contributed to the development of the esophageal candidiasis. 

A 2018 study [7] reported that diabetes mellitus was present in 30% of the cases among 20 HIV-negative patients with esophageal candidiasis, probably associated with consequent immunosuppression and esophageal motility dysregulation and subsequent stasis of its content [7,8]. However, it isn't clear that this complication may arise in long-term, well-controlled diabetic patients.

As for the proton pump inhibitors, the association between their use and the development of esophageal candidiasis is still controversial and not well established. A study published in 2018 suggests that the use of proton pump inhibitors may be associated with the development of esophageal candidiasis in immunocompetent patients [9]. In this study, approximately three-quarters (74%) of the participants were found to be taking a proton pump inhibitor at the time of diagnosis. Treatment with a proton pump inhibitor may reduce gastric acid secretion, potentially leading to a relative state of hypochlorhydria [10]. In another study published in 2015, an association of esophageal candidiasis with proton pump inhibitors treatment as a cause was not found [11]. This drug class is widely prescribed, and only a small fraction of patients develop esophageal candidiasis. Further controlled studies may shed additional light on this potential association.

Regarding inhaled corticosteroids, oropharyngeal candidiasis is a known possible consequence in some patients, particularly elderly patients with other risk factors for candidiasis [12]. In a systematic review of trials of inhaled corticosteroids used to treat chronic pulmonary obstructive disease, the risk of oropharyngeal candidiasis was increased among inhaled corticosteroids users compared to placebo (OR: 2.65, 95% CI; 2.03-3.46; 5,586 participants) [13]. Esophageal candidiasis is a less commonly reported complication in users of inhaled corticosteroids; however, some case reports have suggested this association [3,14]. The use of a different device of dry powder budesonide may favor esophageal drug deposition and Candida spp. infection [3]. Large volume spacer devices may protect against oropharyngeal candidiasis and possibly esophageal candidiasis by reducing the amount of drug deposited in the oropharynx. In addition, rinsing the mouth and throat with water and expectorating the rinsate after the use of all forms of inhaled corticosteroids may have a preventive effect [7]. We decided to discontinue budesonide in this patient since there was no evidence of overlap with asthma, frequent exacerbations, or significant eosinophilia in his medical history [15].

## Conclusions

The esophageal candidiasis of this patient was possibly caused by diabetes mellitus and iatrogenic risk factors. Diabetes can cause immunosuppression, especially if poorly controlled, predisposing to infections by various agents, such as fungi. Clinicians need to keep patients aware of this risk. Iatrogenic immunosuppression may occur with corticosteroids, even when used topically. It is relevant that pharmacists and clinicians develop efforts to minimize its misuse. Proton pump inhibitors may cause a hypochlorhydric state, which weakens one of the gastric mucosa defense mechanisms. Clinicians should keep in mind that these drugs may increase the risk of infections. This case highlights the deleterious effects of polypharmacy and underlines the importance of a proper therapeutic review in every appointment in order to avoid long-term prescriptions without clinical indication and subsequent adverse effects.
